# Norms in action? On the channels through which Poland’s historical partitions may still contribute to divergent educational achievements in the country’s regions

**DOI:** 10.1371/journal.pone.0289686

**Published:** 2023-08-04

**Authors:** Mikołaj Herbst

**Affiliations:** Centre for European Regional and Local Studies, University of Warsaw, Warszawa, Poland; University of Milano–Bicocca: Universita degli Studi di Milano-Bicocca, ITALY

## Abstract

The goal of this paper is to better understand the importance of a long-erased border between two empires that once partitioned Poland and its ongoing effect on regional educational achievement. Previous research has indicated that inherited norms towards education may explain the observed gap in the achievement of students in the former Austrian and Russian partitions of Poland. Findings suggest that although a gap in favour of the former Austrian partition does indeed exist in four school subjects, there is no convincing evidence of the causal effect of historically rooted norms on the achievements of today’s students. If such an effect exists, it is probably indirect and works through the accumulated educational attainments of adult generations and quality of instruction rather than by directly influencing the attitudes of today’s students.

## Introduction

Present-day differences between territories are sometimes difficult to explain by referring only to the contemporary factors that may have caused them. Instead, these discrepancies seem to reflect divisions created by long-forgotten borders between territories that are now constituent parts of the same state. The goal of this paper is to better understand the impact, if any, on regional differences in educational achievement today of a long-erased border between two empires that once partitioned Poland, namely, the 19^th^-century border between the Austrian partition of Poland (hereafter, the Austrian partition) and the territory controlled by the Russian empire (hereafter, the Russian partition). Previous research [1,2] identified inherited norms towards education as a plausible explanation for the observed gap in educational achievement between the two partitions. In this context, the existing norm towards education is understood as the attitude of contemporary students and their families towards schooling, which is based on confidence in the formative role of the school and the belief that education is an investment that will eventually pay off in either pecuniary or non-material terms. However, the evidence used to support the hypothesis that the gap between educational achievements on either side of the former border is due to a social norm was rather indicative and sometimes speculative; thus, it invited further research.

The main research question of the present study is therefore as follows: what are the exact channels through which social norms have affected educational outcomes in two of Poland’s historical partitions? In particular, this investigation brings us closer to understanding whether the observed differences in educational attainment should be attributed to a direct influence of social norms on students’ efforts and commitment to learning or whether we should think of a mediated and staggered impact–one contributing over decades to the divergent levels of more obvious, contemporary inputs into the educational quality in the two regions. Naturally, a sharp separation of direct and indirect effects of norms in an empirical study is not feasible. However, by adopting a nuanced methodological approach and using previously unavailable data, I provide new evidence and shed more light on the role of historical partitions in determining present-day educational quality. Although the present study confirms the existence of a gap in favour of the Austrian partition in terms of achievement in four different school subjects, no strong evidence is found to support the hypothesis of a direct influence of social norms on educational achievement. In this sense, the results of the analysis are not fully consistent with the interpretation presented in previous studies on this issue.

In terms of methodological approach, I propose several enhancements to previous studies investigating the persistent effect of the partitions on today’s gap in student achievements between the regions of Poland. All previous studies have relied on educational achievement statistics aggregated to the level of municipal averages [1–4]. The limited number of observations available seriously limited the statistical significance of the results and the possibility of pursuing more detailed research questions. Moreover, studies typically focused on achievements in mathematics or combined scores in mathematics and science while ignoring those in humanities. Finally, previous studies on this topic were carried out when certain auxiliary data on local educational policies and parental choices with respect to children’s education were not accessible. As such data are now available, new research strategies can be applied to more effectively investigate the relationship between social norms and education.

In the present study, achievements are measured at the school, rather than the municipal, level, thus increasing the number of observations and the statistical reliability of results. Furthermore, I go beyond using a single measure of achievement on the basis that if the divergence of educational outcomes between regions is determined by cultural norms towards education, then it should manifest itself at different stages of students’ educational careers and with respect to different subjects taught at school. However, depending on their nature, these norms may also have distinct effects on students’ attitudes towards particular subjects.

Importantly, while I use several alternative measures of educational achievements, they all pertain to the achievements of the same group of students, and this allows direct comparisons without possible bias due to the change in the composition of the sample.

I also test the largely overlooked hypothesis that the observed gap in achievements may reflect a difference in the quality of instruction that is uncaptured by the measurable inputs. Thus, I also apply a value-added approach using students’ test scores from the earlier educational stage.

Finally, I address the norm towards education more directly than in previous studies, using newly available data on the voluntary enrolment of six-year-old children in schools collected during the unsuccessful attempt made between 2009 and 2016 to lower the age at which children start school in Poland.

The remainder of this paper is organised as follows. Section 2 reviews the literature on how former institutions may affect different aspects of socioeconomic development, including education. Section 3 introduces the historical background of the research, explaining the link between the history of Poland in the period 1795–1918 and the institutional diversity of today’s Polish regions. This section also reviews some earlier studies on the effect of the partitions on educational quality in Poland. Section 4 describes the identification strategy, including the model specification and data used in the analysis. It also explains in detail the role of alternative specifications and the sensitivity analysis I use. Section 5 presents the results of estimations, starting with a replication of Bukowski’s [2] work that draws on more detailed data, before developing alternative specifications. Section 6 summarises the evidence in an attempt to answer the main research question, namely, whether social norms are directly responsible for the observed gap in the educational achievements of students from two historical partitions of Poland.

## Literature review

Differences in the historical development of institutions may be responsible for the divergent development of countries and the regions within them. In their seminal work, Acemoglu et al. [5] argue that the rise of Western Europe from the 15^th^ century was largely due to the strength of domestic institutions predating the era of Atlantic trade. The rise of European empires in the following centuries led to the emergence of a colonial order. Its institutions proved, in turn, to have long-term effects on the colonised regions, contributing to the persistence of socioeconomic differences between now-independent countries with divergent colonial experiences [6–9].

History manifests itself through economic indicators, but also in people’s attitudes and the choices they make. Norms are persistent because they are transmitted between generations in the process of raising children or within social groups [10,11]. However, although the mobility of people contributes to the mixing of different cultures, the territorial anchoring of norms has been strong over the centuries, as studies on Italy [12,13], Germany [14], Poland [15] and China [16] show.

### Possible historical reasons for divergent educational outcomes

Like many other aspects of socioeconomic life, education has been studied in the context of the institutional differences that emerged as a consequence of the colonial division of the world. Research has shown that former British and French colonies have tended to invest more in education than other once-colonised nations since gaining independence [17]. However, the impact of the institutional approach in the early colonial era on subsequent socioeconomic development can be observed even within a single former colonial empire. For example, differences in early investments in human capital and infrastructure between districts in former French West Africa are still reflected in the current performance of these districts concerning education [18].

The projection of historical borders on the maps of educational quality is observed on every continent. In Italy, a country that was unified in the mid-19^th^ century, academic achievement is much higher in the north, in line with differences in per capita income [19]. The United States also exhibits significant inequality in achievement between the northern and southern states, again showing that students in richer regions tend, when family variables are controlled, to have higher education achievements [20].

The positive correlation between the level of economic development of a territory and its educational performance may be considered in the context of the formation of public education systems in the 19^th^ century, which happened earlier in industrialised countries than in agricultural areas. According to some interpretations, physical capital accumulation in the process of industrialisation enhanced the importance of human capital in production and generated incentives for capitalists to support the provision of public education for the masses, triggering the demise of the existing class structure [21]. Other studies, however, point to broader cultural models as determinants of the demand for education in European countries. Chauvel [22] notes that there are three large models for the social structure–the German, the Romance, and that of the less developed Catholic countries. The German model is characterised by limited stratification and the low value of formal education, while the Catholic model involves a strong link between education and socioeconomic status.

The sociological and economic literature indicates that the most likely channels through which historical processes may affect today’s academic performance are the reproduction of parental educational attainment [23,24], quality of instruction at schools [25,26], and individual motivation to study resulting from either the perceived monetary value of education or societal norms towards it [27–30]. The first two factors refer to the typical components of what economists call ‘the education production function’ [31,32]. According to this concept, the achievements of students are determined by their level of family human capital and the level of school resources. If, for historical reasons, these are higher in one region than in another, the uneven achievements of students can nonetheless be ascribed to the persistent effects of institutional differences. However, in such cases, the effect of historical experience on present educational outcomes is indirect and realised through present-day differences in standard educational inputs. This explanation differs from that suggesting that the educational gap between historical regions can be explained directly by the existence of diverse norms towards education among contemporary students, that is, by norms that were shaped in the past and passed down through subsequent generations.

In terms of individual motivation, the norm towards education can be related to a student’s identity, their exposure to social pressure, and the perceived importance of education in realising life projects. Depending on which channel is dominant, the attitude towards education may persist between generations due to parental efforts, social norms prevailing in the neighbourhood, and peer pressure experienced in schools [29,33,34].

Finally, the attitude towards education may have more to do with trust in institutions in general than in schooling in particular. The relevance of trust in institutions, as an important category differentiating the societies in Central and Eastern Europe, has already been demonstrated by Becker et al. [35], who found that historical Habsburg affiliation (as in the Austrian partition) relates to an increased level of current general trust and reduced corruption in the courts and police.

### Historical partitions, socioeconomic development, and educational performance in Poland

Poland is a valuable case for research on the impact of history on socioeconomic development. The first reason for this is its turbulent history. Between 1772 and 1795, following a series of military defeats, Poland was divided by its three neighbouring powers, Russia, Prussia, and Austria. This division took place in three stages and, in 1795, involved the final remnant of independent Polish territory. Until the reunification of Poland in 1918, the three regions were exposed to very different political and administrative cultures and experienced very different rates of economic growth, with the territories under Russian rule being generally less advanced economically and lagging in terms of the development of modern social and political structures.

Indeed, research shows that territorial differences in many socioeconomic phenomena in today’s Poland clearly reflect the historical partitions. For example, the former borders are reflected in the political choices of Polish voters. Voters in the post-Prussian territory, and in the west of Poland generally, tend to be more pro-European and liberal than those in the post-Russian region, which, in turn, is more conservative [36–38].

Some studies on the persistent traces of the empires that previously ruled Polish territory refer directly to education and student achievements. All these studies were carried out after the introduction of countrywide standardised tests in Poland in 2002 and used the test scores as the main endogenous variable. Nonetheless, these studies are quite differentiated in their approaches and methods. The earlier works, which used the first round of school tests, relied mostly on descriptive and cartographic analyses [39,40]. Later attempts employed cross-section regressions [3,4], fixed effects models [1], and geographical regression discontinuity design (GRDD) [2]. All analyses to date have been carried out on data aggregated to the municipal or an even higher level.

Since the paper by Bukowski [2] was a key inspiration for this study, it deserves a short paragraph. The author uses municipal data on the average results of the 9^th^-grade test in mathematics and science to demonstrate (in the RDD framework) the persistent gap in educational achievements between the former Austrian and Russian partitions. In contrast, the author found no difference in educational achievements between the former Russian and Prussian territories. Bukowski argues that the historical foundation of individual identity might be crucial to understanding this phenomenon. In the Austrian educational system, the positive framing of Polish identity (including the use of Polish as the language of instruction) might have created a positive social norm regarding education among Poles. Conversely, in the Russian system, Polish identity had a negative framing and thus might have led to either a neutral or a negative social norm. Bukowski finds partial confirmation of this hypothesis in general surveys containing education-related questions.

Summarising the existing research on the geography of educational quality in Poland, most authors observe that the former Austrian partition outperforms other regions of Poland in terms of academic achievements, as measured by the standardised school tests [1–4,39]. Some studies also acknowledge that the western territories of Poland, formerly under Prussian rule, perform surprisingly poorly given their dominant role in the domestic economy [1].

Although the higher quality of education in the former Austrian partition is commonly recognised, there is little understanding of the mechanisms behind this phenomenon. The channels whereby regional differences in educational achievement have persisted in Poland offer a challenging and unsolved research puzzle. Past studies elaborate on possible explanations, pointing out regional differences in monetary returns to education [1,3], the positive framing of Polish identity in 19^th^-century Austrian schools, as opposed to schools in other partitions [2], and the regionally uneven trust in the formative role of education, beyond its advantages as an ‘investment’ [41].

The major problem with the existing evidence on a distinct social norm towards education in southern Poland is that it is not directly linked to data on student achievement; thus, it is difficult to assess the extent to which the norms explain the observed achievement gap between the former partitions. The attempts to verify the causal relationship amount to either qualitative insights or indirect quantitative analyses based on circumstantial evidence.

It is important to bear in mind that even if the partitioning of Poland resulted in divergent norms towards schooling in the 19^th^ century, this does not necessarily mean that these norms must have affected the educational choices of subsequent generations. After all, individuals in each generation since the partitions were affected have been making concrete educational decisions that, in turn, have incrementally led to differences in the resources that shape students’ achievements and the norms they profess today. Thus, it is not only easy to mistake cause and effect but also to overestimate the direct contribution of norms. Indeed, the achievement gap may reflect entirely typical determinants of academic performance, such as parental educational attainment or a dominant career model in the peer group. Although these factors that may have been gradually determined by historically driven norms, but their effect on contemporary student achievements is straightforward and not strongly related to students’ beliefs.

In sum, according to the existing literature, there are several channels through which the historical divisions of Poland may cause a discrepancy in student achievements across the former partitions today despite the fact these territories were recovered by Poland a century ago. Some channels are rather direct in nature: that is, better access to education and its positive framing in the 19^th^ century might have led to higher educational attainment in consecutive generations. The better achievements of today’s students may therefore be due to the well-studied phenomenon of the reproduction of parental socioeconomic status.

Other potential channels are, however, less obvious: the divergent historical experience of Poland’s partitions might have contributed to the development of a different social norm towards education–one that manifests itself in how much parents, independent of their own educational credentials, trust educational institutions, how much effort students exert while taking low-stakes tests at school, or, finally, the importance attributed to education by local politicians and administrators and the portion of public resources spent on it.

A useful analogy addressing the nature of such ‘norm-mediated’ effects of past events can be found in the literature on the evaluation of public policies. Angelucci and Di Maro [42] distinguish between the ‘treatment’ effect and different kinds of ‘spillover effects’ following any intervention. Given that, in our case, treatment refers to territorial partitions that ceased to exist just over a century ago, all the present consequences should be considered in terms of spillover effects. Importantly, Angelucci and Di Maro clearly distinguish between spillovers based on direct social interactions (which, in the current study, may include the intergenerational transmission of educational attainment) and ‘context equilibrium effects’, which arise when an intervention alters behavioural and social norms.

An additional explanatory lead that has been largely unexplored in previous research is related to differences in the quality of school instruction. It is worth emphasising that compulsory schooling in the Austrian partition was introduced in 1869 (as in the rest of the Austrian empire), while education in the former Russian partition did not become compulsory until 1918, when Poland regained its independence. The Austrian partition not only had a longer tradition of schooling but also more experience in teacher training, which took place at universities in Lviv and Krakow. Unlike in the Russian partition, teachers in Galicia (the historical name of the region under Austrian control) were recruited from the Polish population, as Polish was the main language of instruction [43]. All these factors may have contributed not only to divergent norms towards education in society but also to a better quality of schooling in the former Austrian partition.

In the next section I address all these channels while presenting a conceptual framework of sensitivity analysis.

## Conceptual framework and data

### Measuring the effect of the former borders

As the first phase of my work, I measure the achievement discrepancies at the former border between the Austrian and Russian partitions. Like Bukowski [2], I use a geographical regression discontinuity design (GRDD) [44–46]. In this kind of quasi-experimental approach, it is assumed that location (living, attending school) on one or the other side of the border is random. The ‘treatment’ relies on the exposure of individuals to certain cultural and institutional arrangements due to their location on one side of the geographical boundary, while the location on the other side of the border implies the lack of treatment.

The basic equation estimated within the RDD is:

$${Sms}_{i}=\beta_{0}+\beta_{1}D_{i}+\beta_{2}{dist}_{i}+\beta_{3}D_{i}{dist}_{i}+[\beta X_{i}]+\varepsilon_{i}$$ (1)

*Sms*_*i*_ represents the dependent variable, namely, the average combined score in mathematics and science achieved in the 9^th^-grade test in 2015 in middle school *i*. In this first specification, for the sake of comparability with Bukowski’s [2] research, I focus on achievements in mathematics and science, leaving aside the results in other subjects.

*D*_*i*_ is a treatment variable (denoting the former partition), and *dist*_*i*_ is the distance from the historical border. I estimate Eq (1) for the schools located within a 50 km bandwidth from the border between the former Austrian and Russian partitions.

In further specifications, to improve the precision of the estimated RDD treatment effect, I include covariates, namely, the gender balance and percent of dyslexic students in school *i*. When testing the hypothesis of human capital reproduction, I include the percentage of degree holders and the scale of income deprivation in the local population. For the sake of the efficiency and comparability of estimators, I use the covariate-adjusted RDD estimator with robust standard errors developed by [47].

**Fig 1:**
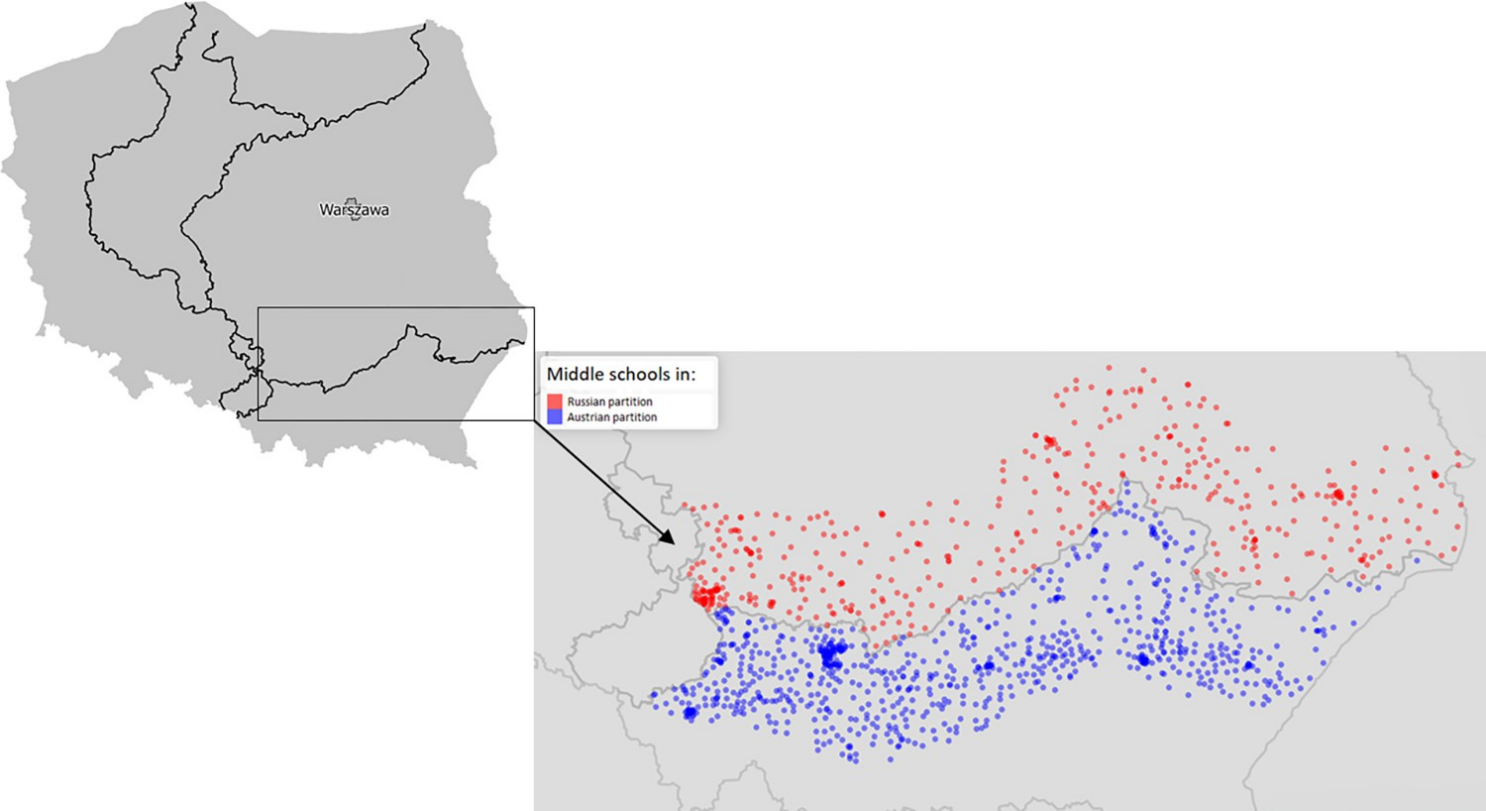
Location of middle schools within a 50 km bandwidth of the historical border between the Austrian and Russian partitions. Source: Author’s own, based on Polish school register (www.dane.gov.pl) and shapefile with a contour map of Poland available at www.geoportal.gov.pl.

The estimation of the RDD model is performed at the school level (each observation in my data refers to one middle school), but it is weighted by school size (number of students); thus, larger schools have a larger impact on the outcomes. The exact locations of schools included in the sample (within a 50 km bandwidth from the border) are shown on the map in Fig 1.

**Fig 2:**
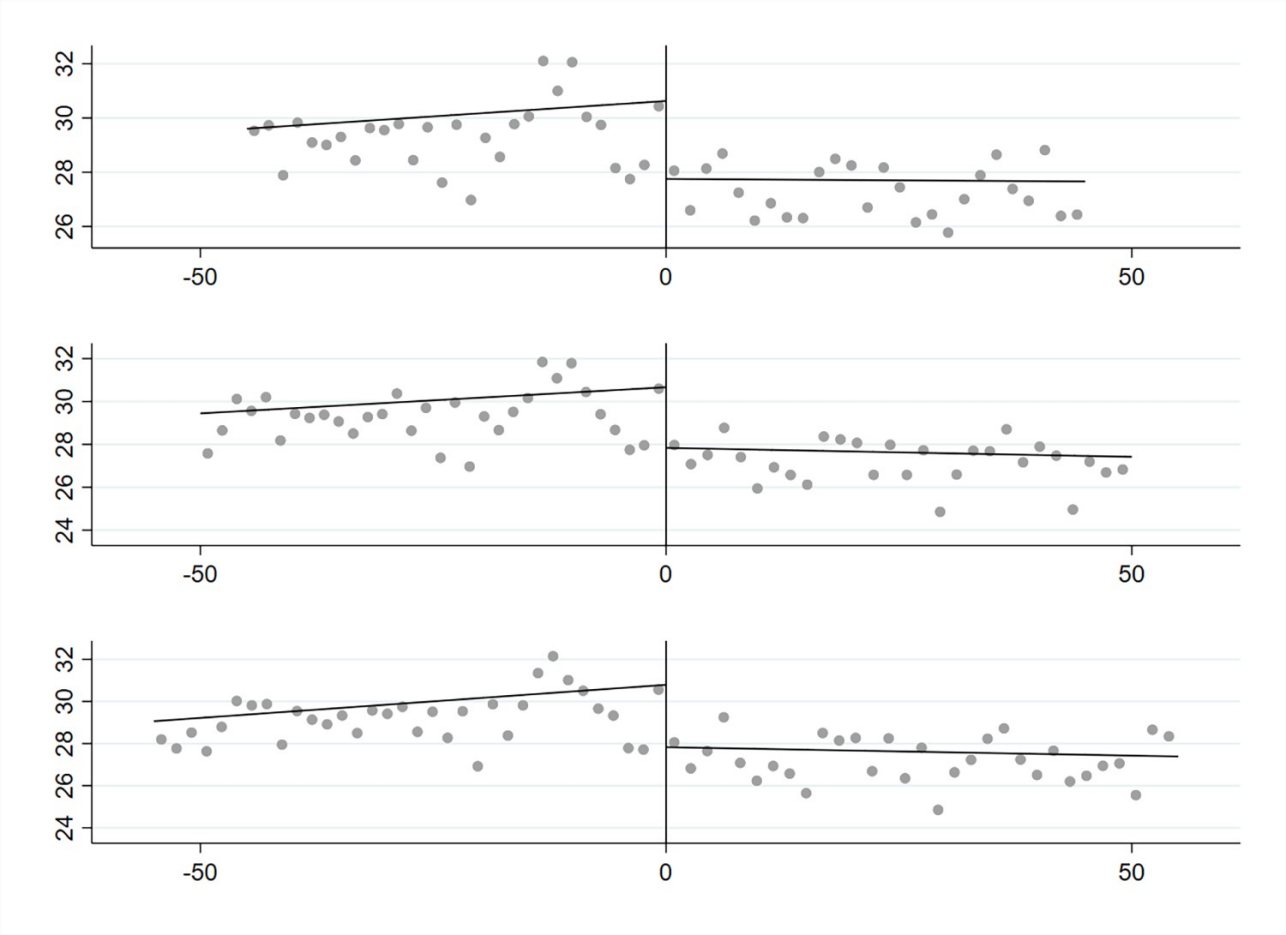
Regression discontinuity at the former border between the Austrian and Russian partitions for the 45 km, 50 km, and 55 km bandwidths.

As measuring the change in levels of student achievement at the former Russian–Austrian border is, to a large extent, a replication of the earlier analysis by Bukowski [2] (although I use school-level data instead of municipality-level aggregates, and a covariate-adjusted estimator in place of a conventional one), I perform only limited diagnostics of the model. Bukowski demonstrated that the results of RDD on the former Russian–Austrian border are largely insensitive to the method of measuring the distance (one- versus two-dimensional), functional form (linear, quadratic, cubic), inclusion versus exclusion of urban municipalities, and inclusion versus exclusion of geographic controls. I supplement these findings by testing different bandwidths of between 45 and 55 km from the border (see Table 2 and Fig 2).

### Understanding the channels through which historical partitions may affect educational achievements, behaviour, and policies

In the further stage of analysis, specification (1) is expanded and then estimated for different alternative dependent variables to test potential explanatory channels. All estimations are performed within the RDD framework with the same bandwidth around the former Austrian–Russian border as applied in the primary estimation.

#### Attitude (norm) towards education

Previous research suggests that the persistence of an achievement gap between Poland’s former partitions may be related to divergent norms towards education that emerged as a consequence of different policies administered by the 19^th^-century empires concerning the cultural and political autonomy of the Polish population [1,2]. Although the difference in attitude towards education is, in fact, observed [41], the direction of causality is not clear. While the higher achievement of students in the Austrian partition may be explained by their positive norm towards education, the norm may also stem from the higher level of human capital.

Norms towards education can manifest in many different ways. Therefore, I use a nuanced strategy of testing, as described below.

#### Testing the partition gap for different school subjects

In the case of the 9^th^-grade test administered in 2015, we can separately measure students’ scores in mathematics, science, Polish, and history. If the gap in educational achievements between former partitions is caused by long-established norms towards school education, it is it is reasonable to assume that students in different partitions score differently in all these subjects. However, the magnitude of the partition effect may not be perfectly symmetrical, depending on the nature of the underlying norm. Bukowski [2] emphasised the positive framing of Polish identity by the Austrian educational system as a potential source of durable advantage of the former Austrian partition in terms of students’ school achievements. If this intuition is correct, one should expect that the gap in achievements in history and Polish would be at least as large as it is for mathematics, or even greater, given that Herbst [41] noticed that parents here tended to care more about the formative role of the school than about the ‘usefulness’ of the skills it teaches.

#### Voluntary enrolment in school as proof of parental trust

Previous studies suggest that the Austrian approach to developing public education in the 19^th^ century may have resulted in a higher level of trust towards educational institutions than that seen in the other two partitions of Polish territory [2,3]. It is, however, difficult to assess trust in education other than by directly asking parents or students about it, although stated preferences are typically biased [48]. In this study, I apply a useful approximation of the revealed, rather than declared, trust towards educational institutions by exploiting the fact that between 2009 and 2016 the Polish government attempted to lower the school starting age from seven to six. In the transitory phase of the programme, parents could decide whether or not to enrol a six-year-old child in school. I consider the choice of earlier enrolment to be a credible indicator of trust in the education system. I then measure the percentage of eligible children enrolled in the first grade in each municipality to approximate the local level of parental trust and use it as the dependent variable in the RDD estimation at the former Austrian–Russian border.

#### More resources spent on schooling?

The uneven importance attached to education can also be seen in the level of resources spent on providing it to the public. Norms may shape policies in this matter, as the decisions taken by the relevant authorities need to follow the preferences of the voters. Thus, one would expect the positive norm to be associated with a higher level of funding. Therefore, I use individual data on the wages of teachers to test whether students’ achievements change at the former border between the partitions. By doing so, I exploit the Polish regulations that require local educational authorities to pay teachers a certain minimum wage (set for each of the four levels of professional advancement) but give them the discretion to set the wages above these minimal levels [49].

#### Better quality of instruction

In the final step of the analysis, I address a hypothesis that has been largely neglected in the earlier research, namely, that the gap in educational achievements observed between the two historical territories is related to the difference in the effectiveness of school instruction (which is not necessarily identical to the difference in resources spent on education). Measuring the effectiveness of schooling has always been problematic because of the obvious difficulty of isolating the contribution of schools from all other factors determining the educational achievements of students. I overcome this problem by applying a value-added approach. As the dependent variable in the analysis refers to student scores at the end of middle school (grade 9), at the right side of the equation I include the average score achieved by the same students at an earlier stage of education, that is, at the end of primary school (grade 6). However, since 6^th^-graders in 2012 were only tested on mathematics and Polish, I constructed a best-matching dependent variable, namely, the combined score from these two subjects as achieved in the 2015 9^th^-grade examination.

The idea behind the value-added approach is that the achievements of students in the 6^th^ and 9^th^ grades are both affected by parental human capital and social norms. There is no reason to believe that social attitudes towards education only matter for one exam. However, it seems reasonable to assume that norms have a stronger impact on achievement at a younger age. Firstly, younger children may spend more time with their parents and thus be influenced by them. Secondly, whereas the results of the 9^th^-grade exam play an important role in secondary school admissions, the 6^th^-grade test is low-stakes. Hence, the effort put in by students may depend on their personal attitude.

Consequently, if the gap between partitions remains significant after controlling for student achievement in grade 6, this suggests that it ‘emerges’ (at least in part) between grades 6 and 9 and has something to do with the quality of educational institutions rather than social norms (which presumably remain constant over such a short period).

#### Control variables to capture the reproduction of socioeconomic status

Typically, student outcomes are strongly correlated with parental education attainment, the latter being approximated by level, number of years in school, or–if available–the actual measure of cognitive skills [32]. Although some of this correlation may result from the intergenerational transfer of norms towards education, having educated parents also provides more ‘direct’ advantages, such as better access to learning resources, direct assistance from family members, or higher pressure to perform well at school. For these reasons, I include a variable describing educational attainment in the municipal adult population in all specifications of the model. As studies show that the mother’s education has a stronger effect on student achievements than the father’s [50,51], I include the percentage of adult females holding a university degree. Additionally, as economic deprivation is considered an important barrier to educational aspirations [52], the percentage of families in the municipality receiving income-based social benefits is also included.

### Data

Table 1 includes descriptive statistics for all the variables used in the analysis, with means and standard deviations computed separately for the former Austrian and Russian partitions as well as for the whole territory within 50 km of the former Austrian–Russian border. The statistics show that average student achievements in all subjects and at both schooling tiers are higher on the Austrian side of the border. The gender balance is similar in the two partitions, but the schools on the Austrian side are significantly larger (76 students, on average, in the final grade vs. 67 in the former Russian partition).

**Table 1:** Descriptive statistics for observations within 50 km of the border.

	Austrian partition		Russian partition		Total		Level	N	Year
	Mean	SD	Mean	SD	Mean	SD			
Score in mathematics (9^th^ grade)	15.24	2.64	13.75	2.02	14.74	2.55	School	1,218	2015
Score in science (9^th^ grade)	14.82	1.96	13.91	1.47	14.52	1.86	School	1,218	2015
Score in Polish (9^th^ grade)	20.63	1.87	19.90	1.56	20.39	1.80	School	1,218	2015
Score in history/civic education (9^th^ grade)	21.47	1.92	20.53	1.61	21.16	1.88	School	1,218	2015
Score in mathematics/Polish (6^th^ grade)	24.08	2.90	22.65	2.22	23.61	2.77	School	1,218	2012
Percentage of boys	50.19	9.23	49.77	8.48	50.05	8.99	School	1,218	2015
Number of students (school size)	75.81	50.29	66.76	39.38	72.81	47.13	School	1,218	2015
Percentage of dyslexic students	13.70	9.95	9.65	8.06	12.36	9.56	School	1,218	2015
Percentage of income-deprived families	5.43	3.06	6.10	3.22	5.65	3.13	Munic.	417	2008
Percentage of degree-holding females	18.43	8.70	16.07	6.15	17.65	8.02	Munic.	417	2011
Percentage of 6-year-olds voluntarily in school	12.81	6.77	13.46	8.60	13.02	7.43	Munic.	417	2013
Wage of novice teacher	2391	367	2428	444	2401	390	Teacher	1,930	2017
Wage of experienced teacher	4667	929	4717	987	4685	952	Teacher	38,505	2017

Dyslexia is more commonly diagnosed on the Austrian side of the border. The Austrian partition also has a slightly lower level of income deprivation (5.4% vs. 6.1), and visibly higher educational attainment among female adults. However, the voluntary enrolment of six-year-olds in schools is more common in the former Russian territory. The average teacher wage is also higher in the former Russian partition, although in this case, the gap is very small (about 1.5% for experienced teachers and 1% for novice teachers).

In the main part of the analysis, the dependent variable is the school-level mean from test scores achieved by students in 9^th^ grade in the final, compulsory examination taken by all students graduating from middle schools in 2015. However, for the sake of comparability with previous research or with students’ earlier achievements, alternative definitions of student achievement are applied in different sections. Initially (Table 2), I use the combined score in mathematics and science (to mimic the approach of Bukowski [2]). Table 3 presents the four different dependent variables, namely, the four main subjects covered by the examination: mathematics, science, Polish, and history. Finally, in the value-added specification (Table 5), the dependent variable is the combined score in mathematics and Polish, as these subjects are covered by the test taken in 6^th^ grade, the results of which are included as an explanatory variable. All data on students’ test scores is administered by Poland’s Central Examination Committee (CKE).

**Table 2:** RDD point estimates at the former Austrian–Russian border for different bandwidths.

Covariates	Conventional	Bias-corrected	Robust	Bandwidth	N
No	-0.687***	-0.412***	-0.412**	45	1,137
	(0.146)	(0.146)	(0.185)		
	-0.673***	-0.530***	-0.530***	50	1,247
	(0.142)	(0.142)	(0.18)		
	-0.671***	-0.573***	-0.573***	55	1,334
	(0.139)	(0.139)	(0.176)		
+ Gender balance, percentage of dyslexic students	-0.526***	-0.335**	-0.335*	45	1,137
	(0.144)	(0.144)	(0.187)		
	-0.512***	-0.436***	-0.436**	50	1,247
	(0.139)	(0.139)	(0.181)		
	-0.510***	-0.468***	-0.468***	55	1,334
	(0.136)	(0.136)	(0.175)		
+ Percentage degree holders (females), the scale of income deprivation	-0.413***	-0.330**	-0.330*	45	1,137
	(0.138)	(0.138)	(0.188)		
	-0.413***	-0.363***	-0.363**	50	1,247
	(0.133)	(0.133)	(0.181)		
	-0.416***	-0.374***	-0.374**	55	1,334
	(0.129)	(0.129)	(0.175)		

Standard errors in parentheses.

* *p* < 0.10

** *p* < 0.05

*** *p* < 0.01.

Dependent variable is the combined test score in mathematics and science.

**Table 3:** RDD point estimates at the former Austrian–Russian border by subject.

Dependent variable	Covariates	Conventional	Bias-corrected	Robust
Mathematics	No	-0.706***	-0.535***	-0.535***
		(0.14)	(0.14)	(0.179)
Polish		-0.549***	-0.551***	-0.551***
		(0.15)	(0.15)	(0.212)
History		-0.525***	-0.404***	-0.404**
		(0.146)	(0.146)	(0.2)
Science		-0.586***	-0.491***	-0.491***
		(0.145)	(0.145)	(0.186)
Mathematics	+ Gender balance, percentage of dyslexic students	-0.548***	-0.442***	-0.442**
		(0.137)	(0.137)	(0.18)
Polish		-0.393***	-0.450***	-0.450**
		(0.144)	(0.144)	(0.211)
History		-0.352**	-0.304**	-0.304
		(0.14)	(0.14)	(0.193)
Science		-0.431***	-0.402***	-0.402**
		(0.142)	(0.142)	(0.185)
Mathematics	+ percentage of degree-holding females and scale of income deprivation	-0.452***	-0.371***	-0.371**
		(0.131)	(0.131)	(0.181)
Polish		-0.303**	-0.380***	-0.380*
		(0.138)	(0.138)	(0.205)
History		-0.254*	-0.233*	-0.233
		(0.132)	(0.132)	(0.188)
Science		-0.334**	-0.330**	-0.330*
		(0.137)	(0.137)	(0.184)

Standard errors in parentheses.

* *p* < 0.10

** *p* < 0.05

*** *p* < 0.01.

Bandwidth = 50 km, N = 1247.

The results presented in Table 4 refer to three alternative dependent variables that do not directly measure student achievements but are likely to be shaped by local norms towards education. The first two are the wages of (respectively) novice and experienced teachers working in public primary schools. Novice teachers are intern educators, typically in their first year of employment at a school. The term ‘experienced teachers’ refers to those who hold the professional degree of ‘licensed teacher’, which, according to the regulations of the Teacher’s Charter Act, can be achieved after a minimum of 10 years’ work.

**Table 4:** RDD point estimates at the former Austrian–Russian border for alternative dependent variables.

Dependent variable	Conventional	Bias-corrected	Robust	*N*
Wage of novice teacher	0.116	0.267**	0.267	1,930
	(0.123)	(0.123)	(1.26)	
Wage of experienced teacher	-0.115***	0.028	0.028	38,409
	(0.0203)	(0.020)	(0.032)	
Percentage of voluntarily enrolled 6-year-old students	0.0258	0.0268	0.0268	417
	(0.022)	(0.022)	(0.034)	

Standard errors in parentheses.

* *p* < 0.10

** *p* < 0.05

*** *p* < 0.01.

Bandwidth = 50 km.

I use data for individual teachers as extracted from the System of Information on Education (SIO), a database administered by Poland’s Ministry of Education. The third dependent variable is the percentage of six-year-old children enrolled voluntarily in the first grade of primary school for which data are available at the municipality level. There are 2,477 municipalities in Poland. These self-governing administrative units are responsible for providing many public services, including primary and lower secondary schooling. The data were extracted from the Local Data Bank (BDL) provided by Poland’s Central Statistical Office (GUS).

Some of the model specifications in this study include additional covariates to obtain more precise point estimates or for the sake of sensitivity analysis. The school-level proportion of students by gender and the share of dyslexic students are extracted from the CKE examination data. The percentage of female adults holding a degree comes from the national census (2011) and is available at the municipal level. Finally, the percentage of families receiving social benefits (2008), used as an approximation of income deprivation, is derived from the POMOST system used by municipalities to administer their social assistance programmes.

Importantly, the variables available at the municipal level characterise the whole population of the municipality, not just the parents of the students present. Although this may be considered a limitation, it is worth remembering that graduation from middle school was compulsory in Poland’s education system in 2015, implying that the municipal population is a good approximation for the middle school community.

## Results

### The gap at the border as estimated through GRDD

A graphical representation of the effect of the former partitions on student achievements is shown in Fig 2. For the sake of consistency with Bukowski’s [2] study, I use the combined results in mathematics and science. The figure illustrates the discontinuity for three different bandwidths along the border between partitions: 45 km, 50 km, and 55 km. The outcome basically confirms that students in the former Austrian partition outperform those in the former Russian partition. The gap measured at the border exceeds 2 points at the original grading scale, which is about 0.7 of standard deviation as measured for all schools within the bandwidth.

The results in Table 2 also show that the point estimates of discontinuity are significant for all bandwidths tested, even when the covariates characterising the student body at the school level (gender balance and percentage of dyslexic students) are included. Finally, the gap is significant when measured with both conventional estimators and covariate-corrected robust estimators (although in the latter case, the absolute magnitudes of the coefficients are somewhat lower).

In the last variant, the specification is expanded to include the covariates related to the level of educational attainment among adult females in the municipality as well as the scale of income deprivation proxied by the percentage of families receiving social benefits (see bottom section of Table 2). By including these variables, I intend to verify the extent to which the observed gap between the two partitions may be explained by differences in socioeconomic status in the parents’ generation. A substantial drop in the point estimates compared to earlier specifications would suggest that the intergenerational reproduction of human capital is, in fact, responsible for a large part of the observed discontinuity of school achievements.

In fact, after controlling for the socioeconomic status of the feminine part of the local population, the estimated change in student achievements at the former border between the partitions drops from 0.47 to 0.37 of standard deviation, that is, by approximately 20%. However, the gap is still highly significant. Although higher achievements in the former Austrian partition may, in part, be associated with a higher level of human capital in the parent’s generation, a large part of the effect remains unexplained; thus, other explanatory channels should be tested.

### The partition gap as measured by school subject

The alleged positive framing of Polish identity by schools in the former Austrian partition in the 19^th^ century might have contributed to the positive social attitude towards school education. If we consider this historically determined attitude as a main causal factor behind the observed gap in educational achievements between the partitions, we would expect the difference to exist for all school subjects in which students take tests but to be particularly consistent in subjects related to the cultural and national identity of students. This should draw our attention to Polish and history, with elements of civic education, as these subjects were covered by the standardised examination taken by 9^th^-grade students in 2015. Moreover, unlike mathematics and science, which were also tested, Polish language and history are strongly associated with Polish identity.

Table 3 includes the RDD point estimates for each of the four subjects. As it turns out, with no covariates included in the model specification, the gap at the former border between partitions is significant for all subjects, and its magnitude ranges from 0.4 standard deviation for the outcome in history to 0.55 of standard deviation for the achievement in Polish. For all four subjects, the achievements are higher in the former Austrian partition.

After controlling for the composition of the student group (gender, dyslexia) and for the socio-economic status of the municipal community (see middle and bottom sections of Table 3), all point estimates decrease. The magnitude of this drop is similar to that observed earlier in Table 2. Importantly, however, changes concerning Polish and science are now significant only at the level of 0.1, and the effect on history becomes insignificant. Mathematics appears to be the only subject for which the gap between the former partitions remains significant, at the level of 0.05.

Therefore, the results do not strongly indicate that the identity-driven norm towards education is a determinant in the educational gap between the former Austrian and Russian partitions of Poland. Although the gap is confirmed for all the subjects taken at the 9^th^-grade examination, the effect for mathematics proves to be the most robust to the sensitivity analysis. Moreover, the achievements in history, which one would expect to be most associated with the sense of cultural and national identity, show no significant gap at the former border when covariates are included in the specification.

### Valuing teachers, entrusting children to schools

In this section, I move away from analysing student achievement and explore other dependent variables that may reflect divergent norms towards education on either side of the former partition border. First, I test whether the norms led to different levels of teachers’ wages in the two partitions. As noted earlier in this article, Polish local governments have substantial autonomy in shaping teachers’ wages within their respective territories on top of the minimum requirements set by the state. The existence of the alleged positive norm towards education in the former Austrian partition could thus result in higher teacher wages, as education is supposed to be valued by local voters. I therefore perform the RDD estimation using two dependent variables, the wages for a novice and an experienced teacher, limiting the sample to educators employed at schools within a 50 km bandwidth of the former border between the partitions.

**Fig 3:**
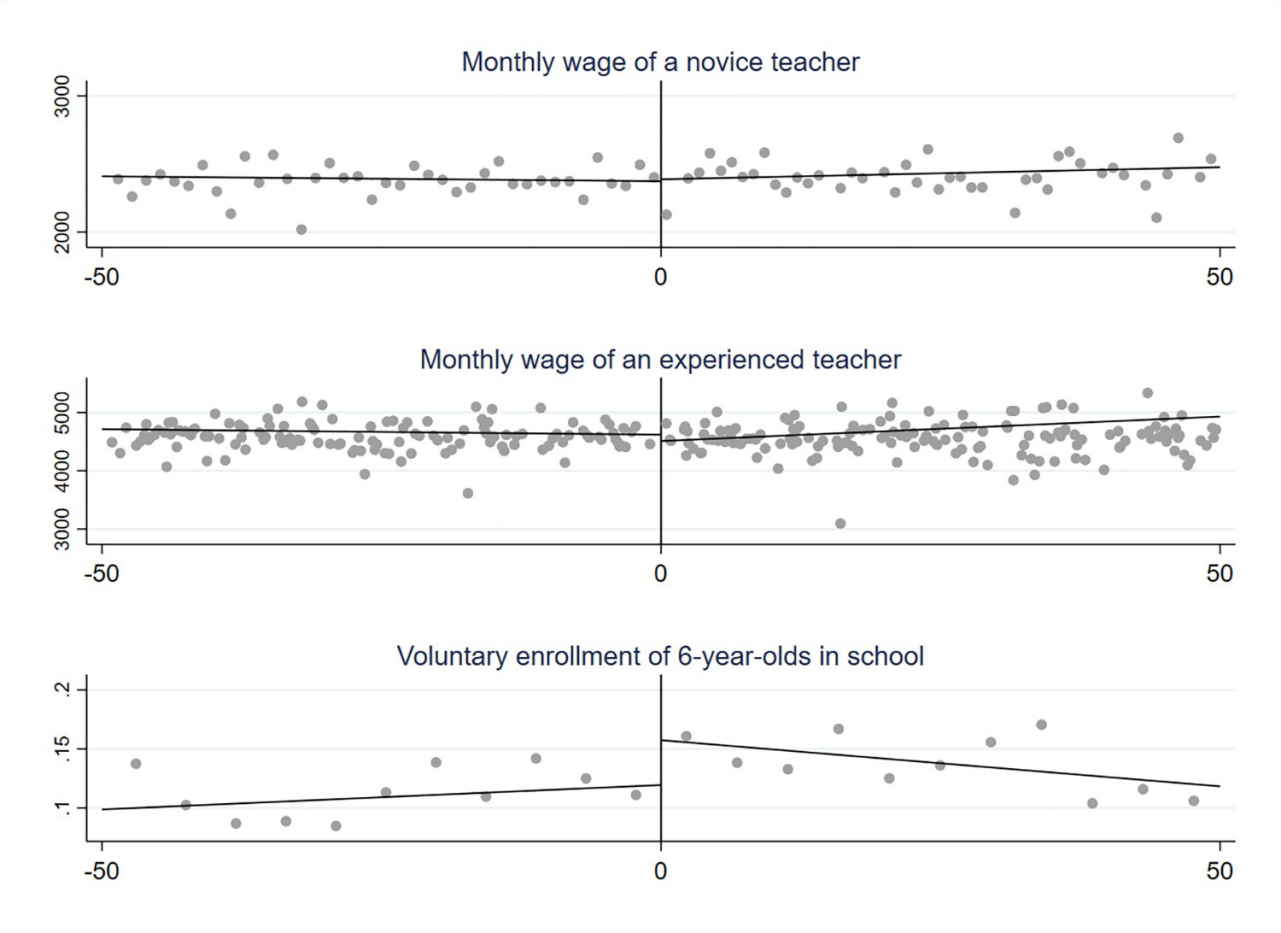
Regression discontinuity at the former border between the Austrian and Russian partitions: Monthly wages of teachers and voluntary enrolment of six-year-old children in schools.

A graphical representation of regression discontinuity for the three dependent variables discussed above is shown in Fig 3. The precise values of RDD point estimates are included in Table 4.

As it turns out, there is no significant difference between the wages of teachers below and above the cut-off point. Although the conventional RDD estimate suggests that the wages of experienced teachers are slightly higher on the ‘Austrian’ side of the border (by 0.1 of standard deviation), this finding is not confirmed when the bias-corrected robust estimators are applied.

Similarly, there seems to be no significant difference between the former Austrian and Russian territories in terms of the voluntary enrolment of six-year-old children in the first grade when the school starting age was changed in Poland.

Therefore, I do not find evidence that the supposedly different norms towards education contributes to either different levels of public spending on schooling or willingness to enrol children in school before it becomes compulsory.

### The value-added specification–are middle schools in the former Austrian partition better?

In the final step of the analysis, I return to using the 9^th^-grade examination score (averaged at the school level) as a dependent variable. However, I expand the original specification of the model by including the earlier achievements of students as an explanatory variable. For each school in the database, I calculate the average score achieved by its students when graduating from primary school, that is, in the 6^th^ grade. The dependent variable is adjusted to match the subjects covered in the earlier examination and is thus based on the combined score in mathematics and Polish.

Overall, as shown in Table 5, the results obtained for this alternative dependent variable are not very different from those in Table 2. However, after the value-added specification is applied (see bottom section of Table 5), the bias-corrected robust estimator becomes insignificant, which implies that the hypothesis that middle schools in the former Austrian partition are of higher quality is not confirmed.

**Table 5:** RDD point estimates at the former Austrian–Russian border–standard versus value-added specification.

Covariates	Conventional	Bias-corrected	Robust
No	-0.674***	-0.569***	-0.569***
	(0.145)	(0.145)	(0.189)
Gender balance, percentage of dyslexic students	-0.509***	-0.468***	-0.468**
	(0.14)	(0.14)	(0.189)
+ percentage of degree holders and scale of income deprivation	-0.410***	-0.394***	-0.394**
	(0.134)	(0.134)	(0.187)
+ value-added	-0.074	-0.159**	-0.159
	(0.068)	(0.068)	(0.099)

Standard errors in parentheses.

* *p* < 0.10

** *p* < 0.05

*** *p* < 0.01.

Dependent variable is the combined test score in mathematics and Polish.

Bandwidth = 50 km, N = 1247.

Unfortunately, since the achievements in the 6^th^-grade examination are available only as a combined score covering two subjects, I cannot assess the value added between the 6^th^ and 9^th^ grades for particular subjects. Such an analysis is worth performing in future studies if more disaggregate data on early achievements become accessible.

## Conclusions

This study confirms the persistence of the achievement gap along the 19^th^-century border between the Austrian and Russian empires on the territory of today’s Poland–a result previously demonstrated by myself [1] and Bukowski [2]. Moreover, it shows that students in the former Austrian partition perform better in each of the four subjects considered, namely, mathematics, Polish, science, and history.

Previous research provides partial evidence supporting the hypothesis that the high achievements of today’s students living in the former Austrian partition are explained by their positive norm towards education, which originated in the liberal policy of the Habsburg Empire that allowed for the cultural autonomy of conquered territories. While this study confirms that the achievement gap between Poland’s historical regions is not fully explained by more standard factors, such as the socioeconomic status of the adult population, I do not find direct evidence that the social norm is the major explanatory factor behind the long-lasting differences in school achievements between the partitions.

After controlling for the basic characteristics of the school communities–gender balance, dyslexia, the educational attainment of adults, and income deprivation within municipalities–the gap in achievements is reduced by roughly 20%. In the case of history and civic education, a school subject particularly associated with national and cultural identity, the gap between the partitions becomes insignificant when covariates are included in the specification. This finding casts doubt on the alleged causal link between the positive framing of Polish identity in the 19^th^ century’s educational system in the Austrian partition and the performance of today’s students in this territory.

Further analysis shows no change at the former border between the partitions concerning two alternative dependent variables: wages paid to teachers and voluntary enrolment of children in school before they reach the compulsory school age. Both these are subject to decisions taken ‘locally’, by either local authorities or parents, and both are arguably related to the social norm towards education. The absence of significant differences between the levels observed on either side of the historical border raises doubts about the decisive role of social attitude towards education in the emergence of the achievement gap between partitions.

Finally, after a value-added approach is applied by controlling for the past achievements of students, it turns out that the size of the achievement gap remains unchanged (indicating the higher value-adding of middle schools on the ‘Austrian’ side of the former border), but the gap becomes statistically insignificant. Therefore, there is no clear evidence that the quality of instruction is higher in the former Austrian partition than in the former Russian one.

What do these results tell us about the transmission of norms towards education as a main channel explaining the ongoing gap between the former partitions? The evidence does not strongly support this line of reasoning, at least not in a way that corresponds to the contextual equilibrium effect defined by Angelucci and Di Maro [42] and that would corroborate the findings of earlier studies [2,41].

Firstly, if the positive attitude towards education in the former Austrian partition originates from the opportunity to cultivate Polish identity and culture under Habsburg rule (as suggested by earlier research), then one could expect that the gap in achievements in history and Polish would be at least as large as that in mathematics and that it would be robust to the inclusion of additional covariates in the specification. Indeed, one could expect it to be even larger, given I noted [41] that parents in the former Austrian partition tended to care more about the formative role of the school than about the ‘usefulness’ of the skills that it teaches. In reality, however, as indicated by the present study, the effect of the Austrian partition on achievements in history and civic education is weaker than it is for other subjects. Secondly, the gap between different historical partitions is not confirmed when alternative measures–ones clearly related to norms towards education, but not referring to today’s achievements of students–are used as dependent variables in the regression discontinuity design.

This study shows that one should be cautious when linking the interregional gap in educational achievements to the contemporary cultural norms of students, especially if many alternative explanations exist. This certainly does not mean that individual attitudes towards education are unimportant in determining students’ success. However, one needs to keep in mind that these attitudes are also shaped by other factors affecting the outcomes of the educational process and subject to reverse causality; that is, a positive norm may simply result from higher educational attainment or a better personal experience at school.

## Supporting information

S1 Data (DTA)
